# Crystal structure of (*E*)-2-[(4-chloro-2*H*-chromen-3-yl)methyl­idene]-*N*-cyclo­hexyl­hydrazinecarbo­thio­amide

**DOI:** 10.1107/S1600536814018509

**Published:** 2014-08-23

**Authors:** Rajeswari Gangadharan, Jebiti Haribabu, Ramasamy Karvembu, K. Sethusankar

**Affiliations:** a Department of Physics, Ethiraj College for Women (Autonomous), Chennai 600 008, India; b Department of Chemistry, National Institute of Technology, Tiruchirappalli 620 015, India; c Department of Physics, RKM Vivekananda College (Autonomous), Chennai 600 004, India

**Keywords:** crystal structure, chromene, hydrazine, thio­amide, cyclo­hex­yl, hydrogen bonds

## Abstract

In the title compound, C_17_H_20_ClN_3_OS, the mean plane of the central thio­urea core makes dihedral angles of 26.56 (9) and 47.62 (12)° with the mean planes of the chromene moiety and the cyclo­hexyl ring, respectively. The cyclo­hexyl ring adopts a chair conformation. The N–H atoms of the thio­urea unit adopt an *anti* conformation. The chromene group is positioned *trans*, whereas the cyclo­hexyl ring lies in the *cis* position to the thione S atom, with respect to the thio­urea C—N bond. In the crystal, mol­ecules are linked by N—H⋯S hydrogen bonds, forming inversion dimers enclosing *R* ^2^_2_(8) ring motifs. The dimers are linked by C—H⋯Cl hydrogen bonds, enclosing *R* ^6^_6_(44) ring motifs, forming sheets lying parallel to (010).

## Related literature   

For the biological properties of thio­semicarbazones, see: Prabhakaran *et al.* (2007 ▶); Kelly *et al.* (1996 ▶); West *et al.* (1993 ▶); Pérez *et al.* (1999 ▶). For their optical properties and applications, see: Tian *et al.* (1997 ▶); Uesugi *et al.* (1994 ▶). For a related structure, see: Jayakumar *et al.* (2011 ▶).
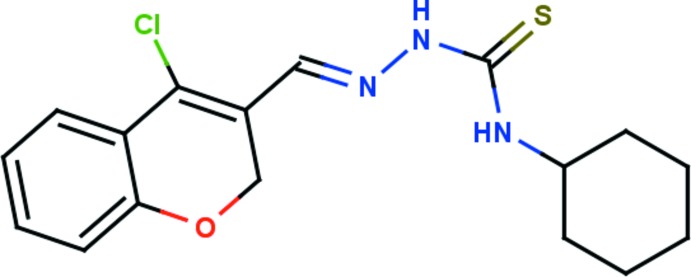


## Experimental   

### Crystal data  

C_17_H_20_ClN_3_OS
*M* *_r_* = 349.87
Orthorhombic, 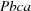

*a* = 12.2857 (12) Å
*b* = 15.3082 (16) Å
*c* = 18.5241 (18) Å
*V* = 3483.9 (6) Å^3^
*Z* = 8
Mo *K*α radiation
μ = 0.35 mm^−1^
*T* = 296 K
0.30 × 0.25 × 0.20 mm

### Data collection  

Bruker SMART APEXII area-detector diffractometer
Absorption correction: multi-scan (*SADABS*; Bruker, 2008 ▶) *T*_min_ = 0.901, *T*_max_ = 0.933
18568 measured reflections
4291 independent reflections
2762 reflections with *I* > 2σ(*I*)
*R*_int_ = 0.033

### Refinement  

*R*[*F* ^2^ > 2σ(*F* ^2^)] = 0.050
*wR*(*F* ^2^) = 0.152
*S* = 1.02
4291 reflections
208 parameters
H-atom parameters constrained
Δρ_max_ = 0.32 e Å^−3^
Δρ_min_ = −0.31 e Å^−3^

### 

Data collection: *APEX2* (Bruker, 2008 ▶); cell refinement: *SAINT* (Bruker, 2008 ▶); data reduction: *SAINT*; program(s) used to solve structure: *SHELXS97* (Sheldrick, 2008 ▶); program(s) used to refine structure: *SHELXL97* (Sheldrick, 2008 ▶); molecular graphics: *ORTEP-3 for Windows* (Farrugia, 2012 ▶); software used to prepare material for publication: *SHELXL97* and *PLATON* (Spek, 2009 ▶).

## Supplementary Material

Crystal structure: contains datablock(s) global, I. DOI: 10.1107/S1600536814018509/su2769sup1.cif

Structure factors: contains datablock(s) I. DOI: 10.1107/S1600536814018509/su2769Isup2.hkl

Supporting information file. DOI: 10.1107/S1600536814018509/su2769Isup3.cml

**Fig. 1.**
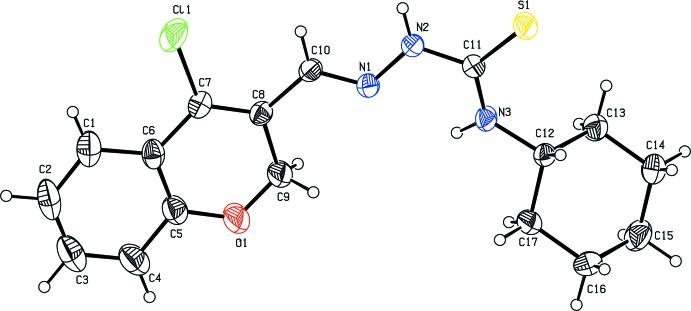
The molecular structure of the title molecule, with atom labelling. The displacement ellipsoids are drawn at the 30% probability level.

**Fig. 2.**
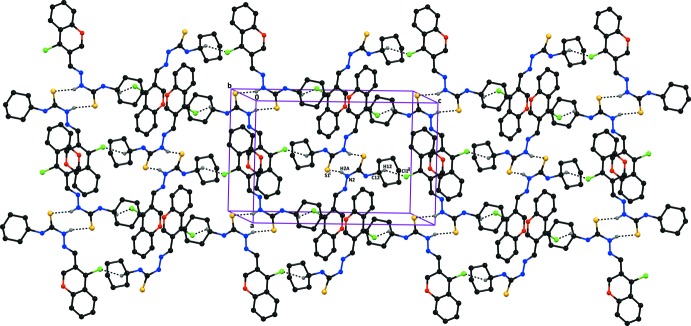
The crystal packing of the title compound viewed along the a axis. The hydrogen bonds are shown as dashed lines (see Table 1 for details; H atoms not involved in hydrogen bonding have been omitted for clarity).

. DOI: 10.1107/S1600536814018509/su2769fig1.tif
The mol­ecular structure of the title mol­ecule, with atom labelling. The displacement ellipsoids are drawn at the 30% probability level.

a . DOI: 10.1107/S1600536814018509/su2769fig2.tif
The crystal packing of the title compound viewed along the *a* axis. The hydrogen bonds are shown as dashed lines (see Table 1 for details; H atoms not involved in hydrogen bonding have been omitted for clarity).

CCDC reference: 1016441

Additional supporting information:  crystallographic information; 3D view; checkCIF report

## Figures and Tables

**Table 1:** Hydrogen-bond geometry (Å, °)

*D*—H⋯*A*	*D*—H	H⋯*A*	*D*⋯*A*	*D*—H⋯*A*
N2—H2*A*⋯S1^i^	0.86	2.73	3.507 (2)	151
C12—H12⋯Cl1^ii^	0.98	2.83	3.689 (2)	147

Symmetry codes: (i) 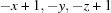; (ii) 
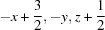
.

## supplementary crystallographic information

### S1. Experimental

An ethanol solution of *N*-cyclohexylhydrazinecarbothioamide (1.736 g, 0.01 mole) was added to a ethanol solution (50 cm^3^) of 4-chloro-2*H*-chromene-3-carbaldehyde (1.94 g, 0.01 mole). The mixture was refluxed for 2 h during which time a yellow precipitate separated out. The reaction mixture was then cooled to room temperature and the precipitate was filtered off. It was then washed with ethanol and dried under vacuum (Yield: 85%). Crystals of the title compound were obtained by slow evaporation of a solution in ethanol.

### S2. Refinement

The positions of the H atoms were localized from difference electron density maps and they were refined as riding atoms: N-H = 0.86 Å, C-H = 0.93 - 0.98 Å, with *U*_iso_(H) = 1.5U_eq_(C-methyl) and = 1.2U_eq_(N,C) for other H atoms.

### Crystal data

**Table:**

C_17_H_20_ClN_3_OS	*F*(000) = 1472
*M**_r_* = 349.87	*D*_x_ = 1.334 Mg m^−^^3^
Orthorhombic, *P**b**c**a*	Mo *K*α radiation, λ = 0.71073 Å
Hall symbol: -P 2ac 2ab	Cell parameters from 2762 reflections
*a* = 12.2857 (12) Å	θ = 2.2–28.3°
*b* = 15.3082 (16) Å	µ = 0.35 mm^−^^1^
*c* = 18.5241 (18) Å	*T* = 296 K
*V* = 3483.9 (6) Å^3^	Block, colourless
*Z* = 8	0.30 × 0.25 × 0.20 mm

### Data collection

**Table:**

Bruker SMART APEXII area-detector diffractometer	4291 independent reflections
Radiation source: fine-focus sealed tube	2762 reflections with *I* > 2σ(*I*)
Graphite monochromator	*R*_int_ = 0.033
ω and φ scans	θ_max_ = 28.3°, θ_min_ = 2.2°
Absorption correction: multi-scan (*SADABS*; Bruker, 2008)	*h* = −15→16
*T*_min_ = 0.901, *T*_max_ = 0.933	*k* = −19→19
18568 measured reflections	*l* = −24→23

### Refinement

**Table:**

Refinement on *F*^2^	Primary atom site location: structure-invariant direct methods
Least-squares matrix: full	Secondary atom site location: difference Fourier map
*R*[*F*^2^ > 2σ(*F*^2^)] = 0.050	Hydrogen site location: inferred from neighbouring sites
*wR*(*F*^2^) = 0.152	H-atom parameters constrained
*S* = 1.02	*w* = 1/[σ^2^(*F*_o_^2^) + (0.0745*P*)^2^ + 0.9377*P*] where *P* = (*F*_o_^2^ + 2*F*_c_^2^)/3
4291 reflections	(Δ/σ)_max_ = 0.001
208 parameters	Δρ_max_ = 0.32 e Å^−^^3^
0 restraints	Δρ_min_ = −0.31 e Å^−^^3^

### Special details

**Table:**

Geometry. All e.s.d.'s (except the e.s.d. in the dihedral angle between two l.s. planes) are estimated using the full covariance matrix. The cell e.s.d.'s are taken into account individually in the estimation of e.s.d.'s in distances, angles and torsion angles; correlations between e.s.d.'s in cell parameters are only used when they are defined by crystal symmetry. An approximate (isotropic) treatment of cell e.s.d.'s is used for estimating e.s.d.'s involving l.s. planes.
Refinement. Refinement of *F*^2^ against ALL reflections. The weighted *R*-factor *wR* and goodness of fit *S* are based on *F*^2^, conventional *R*-factors *R* are based on *F*, with *F* set to zero for negative *F*^2^. The threshold expression of *F*^2^ > σ(*F*^2^) is used only for calculating *R*-factors(gt) *etc*. and is not relevant to the choice of reflections for refinement. *R*-factors based on *F*^2^ are statistically about twice as large as those based on *F*, and *R*- factors based on ALL data will be even larger.

### Fractional atomic coordinates and isotropic or equivalent isotropic displacement parameters (Å^2^)

**Table:**

	*x*	*y*	*z*	*U*_iso_*/*U*_eq_	
C1	1.1410 (2)	0.0421 (2)	0.35313 (15)	0.0735 (8)	
H1	1.1134	0.0105	0.3144	0.088*	
C2	1.2530 (2)	0.0541 (2)	0.36038 (19)	0.0835 (10)	
H2	1.2997	0.0306	0.3259	0.100*	
C3	1.2948 (2)	0.0990 (2)	0.41635 (19)	0.0824 (9)	
H3	1.3698	0.1057	0.4205	0.099*	
C4	1.2276 (2)	0.1348 (2)	0.46699 (17)	0.0790 (8)	
H4	1.2568	0.1662	0.5054	0.095*	
C5	1.11588 (18)	0.12440 (18)	0.46109 (13)	0.0600 (6)	
C6	1.07055 (17)	0.07780 (15)	0.40441 (12)	0.0514 (5)	
C7	0.95198 (17)	0.06950 (15)	0.40340 (11)	0.0485 (5)	
C8	0.88960 (16)	0.10411 (14)	0.45507 (10)	0.0438 (5)	
C9	0.94477 (19)	0.1572 (2)	0.51200 (14)	0.0712 (8)	
H9A	0.9170	0.2164	0.5085	0.085*	
H9B	0.9222	0.1345	0.5585	0.085*	
C10	0.77399 (16)	0.08929 (15)	0.46230 (10)	0.0453 (5)	
H10	0.7364	0.0579	0.4273	0.054*	
C11	0.56710 (15)	0.10953 (15)	0.58999 (10)	0.0444 (5)	
C12	0.59224 (15)	0.17836 (15)	0.71073 (10)	0.0423 (5)	
H12	0.5665	0.1240	0.7327	0.051*	
C13	0.50398 (19)	0.24676 (16)	0.71814 (12)	0.0563 (6)	
H13A	0.4375	0.2255	0.6960	0.068*	
H13B	0.5259	0.2995	0.6930	0.068*	
C14	0.4829 (2)	0.2677 (2)	0.79728 (14)	0.0729 (8)	
H14A	0.4279	0.3129	0.8007	0.087*	
H14B	0.4555	0.2160	0.8215	0.087*	
C15	0.5855 (2)	0.2981 (2)	0.83439 (14)	0.0733 (8)	
H15A	0.6093	0.3527	0.8130	0.088*	
H15B	0.5704	0.3087	0.8850	0.088*	
C16	0.6747 (2)	0.2314 (2)	0.82767 (13)	0.0713 (8)	
H16A	0.6547	0.1794	0.8546	0.086*	
H16B	0.7410	0.2546	0.8486	0.086*	
C17	0.69562 (17)	0.2068 (2)	0.74917 (12)	0.0645 (7)	
H17A	0.7266	0.2566	0.7241	0.077*	
H17B	0.7483	0.1597	0.7474	0.077*	
N1	0.72378 (13)	0.11972 (13)	0.51739 (8)	0.0456 (4)	
N2	0.61712 (13)	0.09549 (13)	0.52482 (8)	0.0481 (4)	
H2A	0.5823	0.0721	0.4895	0.058*	
N3	0.62011 (13)	0.16098 (12)	0.63520 (9)	0.0486 (4)	
H3A	0.6769	0.1870	0.6185	0.058*	
Cl1	0.89480 (7)	0.01105 (6)	0.33357 (4)	0.0936 (3)	
O1	1.05307 (15)	0.16182 (18)	0.51223 (12)	0.1067 (9)	
S1	0.44825 (4)	0.05982 (5)	0.60746 (3)	0.0645 (2)	

### Atomic displacement parameters (Å^2^)

**Table:**

	*U*^11^	*U*^22^	*U*^33^	*U*^12^	*U*^13^	*U*^23^
C1	0.0760 (17)	0.0745 (19)	0.0699 (15)	0.0181 (15)	0.0253 (13)	0.0081 (14)
C2	0.0698 (17)	0.086 (2)	0.095 (2)	0.0314 (16)	0.0410 (16)	0.0311 (19)
C3	0.0520 (14)	0.089 (2)	0.106 (2)	0.0117 (15)	0.0193 (15)	0.039 (2)
C4	0.0504 (13)	0.093 (2)	0.0933 (19)	−0.0055 (14)	0.0029 (13)	0.0141 (18)
C5	0.0483 (12)	0.0669 (16)	0.0649 (13)	−0.0009 (12)	0.0085 (10)	0.0079 (13)
C6	0.0532 (11)	0.0488 (13)	0.0523 (11)	0.0072 (10)	0.0149 (9)	0.0133 (10)
C7	0.0581 (12)	0.0446 (12)	0.0429 (10)	−0.0022 (10)	0.0071 (9)	−0.0021 (9)
C8	0.0481 (10)	0.0453 (12)	0.0381 (9)	−0.0035 (9)	0.0032 (8)	0.0006 (9)
C9	0.0466 (12)	0.100 (2)	0.0675 (15)	−0.0087 (13)	0.0033 (10)	−0.0310 (15)
C10	0.0467 (10)	0.0522 (13)	0.0370 (9)	−0.0054 (10)	0.0004 (8)	−0.0019 (9)
C11	0.0390 (9)	0.0516 (13)	0.0425 (9)	0.0002 (9)	0.0007 (7)	−0.0016 (10)
C12	0.0405 (9)	0.0481 (12)	0.0385 (9)	−0.0048 (9)	0.0027 (7)	−0.0014 (9)
C13	0.0556 (13)	0.0614 (16)	0.0519 (11)	0.0092 (12)	0.0019 (9)	−0.0019 (11)
C14	0.0633 (15)	0.092 (2)	0.0634 (14)	0.0143 (15)	0.0081 (12)	−0.0221 (15)
C15	0.0829 (18)	0.0764 (19)	0.0608 (14)	−0.0063 (16)	0.0083 (13)	−0.0251 (14)
C16	0.0611 (14)	0.097 (2)	0.0556 (13)	−0.0050 (15)	−0.0097 (11)	−0.0245 (14)
C17	0.0412 (11)	0.0898 (19)	0.0625 (13)	−0.0023 (12)	−0.0016 (10)	−0.0232 (14)
N1	0.0417 (8)	0.0540 (11)	0.0412 (8)	−0.0050 (8)	0.0034 (6)	0.0009 (8)
N2	0.0396 (8)	0.0637 (12)	0.0410 (8)	−0.0071 (8)	0.0016 (6)	−0.0058 (8)
N3	0.0423 (8)	0.0598 (12)	0.0436 (8)	−0.0144 (8)	0.0096 (7)	−0.0081 (8)
Cl1	0.0938 (6)	0.1186 (7)	0.0683 (4)	−0.0167 (5)	0.0133 (3)	−0.0505 (4)
O1	0.0485 (10)	0.173 (3)	0.0992 (15)	−0.0152 (12)	0.0062 (9)	−0.0721 (16)
S1	0.0446 (3)	0.0870 (5)	0.0621 (4)	−0.0208 (3)	0.0102 (2)	−0.0205 (3)

### Geometric parameters (Å, º)

**Table:**

C1—C2	1.395 (4)	C11—S1	1.678 (2)
C1—C6	1.397 (3)	C12—N3	1.465 (2)
C1—H1	0.9300	C12—C13	1.514 (3)
C2—C3	1.346 (5)	C12—C17	1.520 (3)
C2—H2	0.9300	C12—H12	0.9800
C3—C4	1.364 (4)	C13—C14	1.523 (3)
C3—H3	0.9300	C13—H13A	0.9700
C4—C5	1.387 (3)	C13—H13B	0.9700
C4—H4	0.9300	C14—C15	1.510 (4)
C5—O1	1.349 (3)	C14—H14A	0.9700
C5—C6	1.386 (3)	C14—H14B	0.9700
C6—C7	1.462 (3)	C15—C16	1.503 (4)
C7—C8	1.336 (3)	C15—H15A	0.9700
C7—Cl1	1.723 (2)	C15—H15B	0.9700
C8—C10	1.445 (3)	C16—C17	1.524 (3)
C8—C9	1.494 (3)	C16—H16A	0.9700
C9—O1	1.332 (3)	C16—H16B	0.9700
C9—H9A	0.9700	C17—H17A	0.9700
C9—H9B	0.9700	C17—H17B	0.9700
C10—N1	1.280 (3)	N1—N2	1.369 (2)
C10—H10	0.9300	N2—H2A	0.8600
C11—N3	1.321 (3)	N3—H3A	0.8600
C11—N2	1.372 (2)		
			
C2—C1—C6	119.7 (3)	C13—C12—H12	108.6
C2—C1—H1	120.2	C17—C12—H12	108.6
C6—C1—H1	120.2	C12—C13—C14	110.75 (19)
C3—C2—C1	121.2 (3)	C12—C13—H13A	109.5
C3—C2—H2	119.4	C14—C13—H13A	109.5
C1—C2—H2	119.4	C12—C13—H13B	109.5
C2—C3—C4	120.3 (3)	C14—C13—H13B	109.5
C2—C3—H3	119.9	H13A—C13—H13B	108.1
C4—C3—H3	119.9	C15—C14—C13	111.2 (2)
C3—C4—C5	119.9 (3)	C15—C14—H14A	109.4
C3—C4—H4	120.0	C13—C14—H14A	109.4
C5—C4—H4	120.0	C15—C14—H14B	109.4
O1—C5—C6	121.4 (2)	C13—C14—H14B	109.4
O1—C5—C4	117.5 (3)	H14A—C14—H14B	108.0
C6—C5—C4	121.1 (2)	C16—C15—C14	111.2 (2)
C5—C6—C1	117.9 (2)	C16—C15—H15A	109.4
C5—C6—C7	117.04 (19)	C14—C15—H15A	109.4
C1—C6—C7	125.1 (2)	C16—C15—H15B	109.4
C8—C7—C6	121.9 (2)	C14—C15—H15B	109.4
C8—C7—Cl1	120.68 (17)	H15A—C15—H15B	108.0
C6—C7—Cl1	117.45 (16)	C15—C16—C17	111.7 (2)
C7—C8—C10	124.63 (19)	C15—C16—H16A	109.3
C7—C8—C9	117.47 (19)	C17—C16—H16A	109.3
C10—C8—C9	117.78 (18)	C15—C16—H16B	109.3
O1—C9—C8	119.0 (2)	C17—C16—H16B	109.3
O1—C9—H9A	107.6	H16A—C16—H16B	107.9
C8—C9—H9A	107.6	C12—C17—C16	112.15 (18)
O1—C9—H9B	107.6	C12—C17—H17A	109.2
C8—C9—H9B	107.6	C16—C17—H17A	109.2
H9A—C9—H9B	107.0	C12—C17—H17B	109.2
N1—C10—C8	119.37 (18)	C16—C17—H17B	109.2
N1—C10—H10	120.3	H17A—C17—H17B	107.9
C8—C10—H10	120.3	C10—N1—N2	116.28 (17)
N3—C11—N2	115.49 (17)	N1—N2—C11	118.36 (16)
N3—C11—S1	125.24 (15)	N1—N2—H2A	120.8
N2—C11—S1	119.25 (15)	C11—N2—H2A	120.8
N3—C12—C13	112.32 (17)	C11—N3—C12	126.76 (17)
N3—C12—C17	107.71 (16)	C11—N3—H3A	116.6
C13—C12—C17	110.98 (19)	C12—N3—H3A	116.6
N3—C12—H12	108.6	C9—O1—C5	123.1 (2)
			
C6—C1—C2—C3	0.5 (4)	C7—C8—C10—N1	174.1 (2)
C1—C2—C3—C4	−0.7 (5)	C9—C8—C10—N1	−1.8 (3)
C2—C3—C4—C5	0.4 (5)	N3—C12—C13—C14	−176.0 (2)
C3—C4—C5—O1	−179.4 (3)	C17—C12—C13—C14	−55.4 (3)
C3—C4—C5—C6	0.1 (4)	C12—C13—C14—C15	57.3 (3)
O1—C5—C6—C1	179.1 (3)	C13—C14—C15—C16	−56.7 (3)
C4—C5—C6—C1	−0.4 (4)	C14—C15—C16—C17	54.5 (3)
O1—C5—C6—C7	−1.3 (4)	N3—C12—C17—C16	176.9 (2)
C4—C5—C6—C7	179.3 (2)	C13—C12—C17—C16	53.6 (3)
C2—C1—C6—C5	0.1 (4)	C15—C16—C17—C12	−53.2 (3)
C2—C1—C6—C7	−179.5 (2)	C8—C10—N1—N2	−173.26 (19)
C5—C6—C7—C8	−0.8 (3)	C10—N1—N2—C11	165.58 (19)
C1—C6—C7—C8	178.8 (2)	N3—C11—N2—N1	13.0 (3)
C5—C6—C7—Cl1	−179.54 (18)	S1—C11—N2—N1	−165.46 (16)
C1—C6—C7—Cl1	0.1 (3)	N2—C11—N3—C12	−172.13 (19)
C6—C7—C8—C10	−171.9 (2)	S1—C11—N3—C12	6.2 (3)
Cl1—C7—C8—C10	6.7 (3)	C13—C12—N3—C11	−81.4 (3)
C6—C7—C8—C9	4.0 (3)	C17—C12—N3—C11	156.1 (2)
Cl1—C7—C8—C9	−177.36 (19)	C8—C9—O1—C5	3.6 (5)
C7—C8—C9—O1	−5.4 (4)	C6—C5—O1—C9	−0.3 (5)
C10—C8—C9—O1	170.8 (3)	C4—C5—O1—C9	179.2 (3)

### Hydrogen-bond geometry (Å, º)

**Table:**

*D*—H···*A*	*D*—H	H···*A*	*D*···*A*	*D*—H···*A*
N2—H2*A*···S1^i^	0.86	2.73	3.507 (2)	151
C12—H12···Cl1^ii^	0.98	2.83	3.689 (2)	147

Symmetry codes: (i) −*x*+1, −*y*, −*z*+1; (ii) −*x*+3/2, −*y*, *z*+1/2.
