# Supplementary figures and images for: Crystal structure of (E)-2-[(4-chloro-2H-chromen-3-yl)methyl­idene]-N-cyclo­hexyl­hydrazinecarbo­thio­amide
Source: Acta Crystallogr Sect E Struct Rep Online. 2014 Aug 23;70(Pt 9):o1039–40. doi: 10.1107/S1600536814018509 (PMC4186115; doi:10.1107/S1600536814018509)

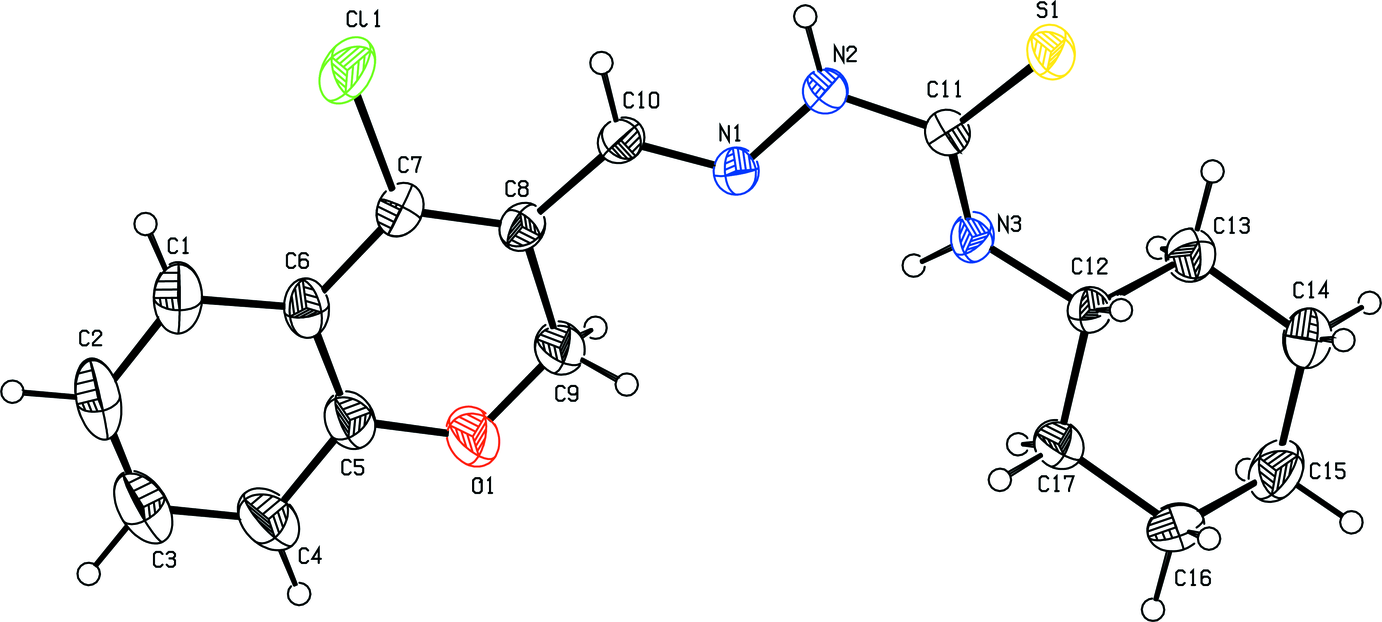

Supplement: Supplementary file 4 [file e-70-o1039-fig1.tif]

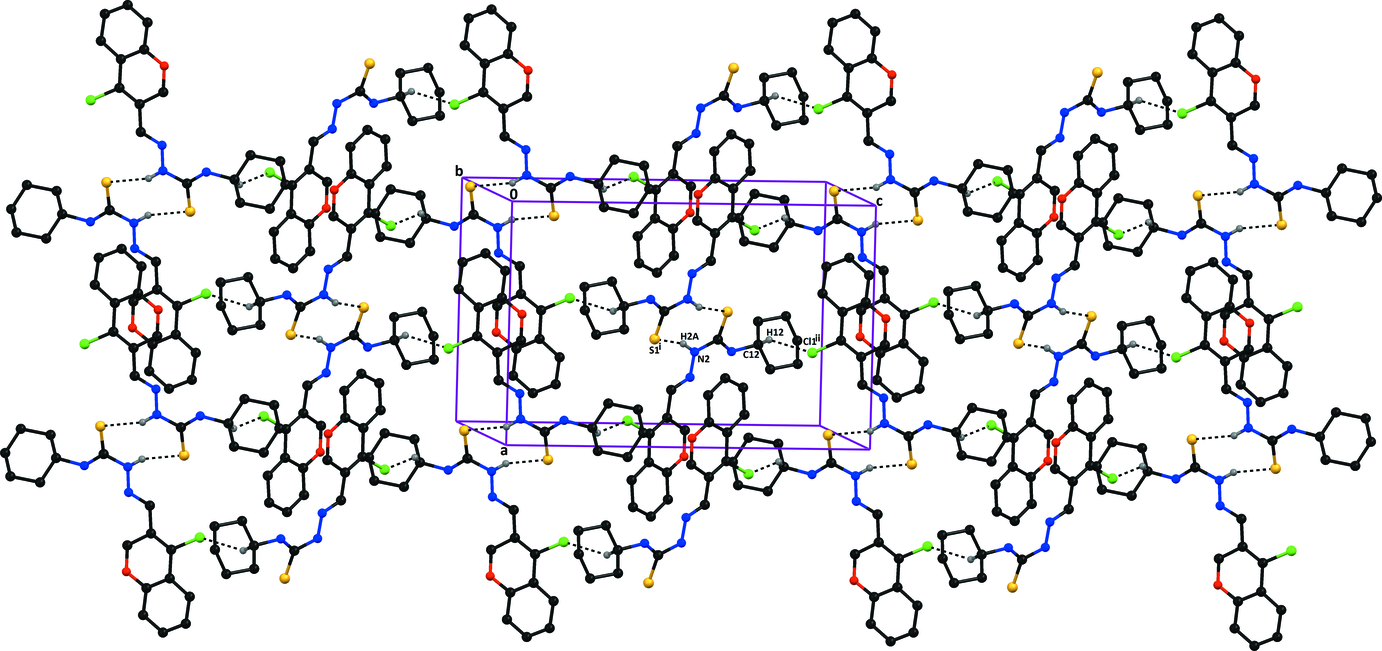

Supplement: Supplementary file 5 [file e-70-o1039-fig2.tif]
